# Bioanalytical method development and validation of HPLCUV assay for the quantification of SHetA2 in mouse and human plasma: Application to pharmacokinetics study

**DOI:** 10.7243/2050-120X-6-2

**Published:** 2017

**Authors:** Ankur Sharma, Elangovan Thavathiru, Doris Mangiaracina Benbrook, Sukyung Woo

**Affiliations:** 1 Department of Pharmaceutical Sciences, College of Pharmacy, University of Oklahoma Health Sciences Center, 1110 N. Stonewall Ave. CPB331, Oklahoma City, Oklahoma 73117-1200, USA; 2 Department of Obstetrics and Gynecology, Stephenson Cancer Center (SCC), University of Oklahoma Health Sciences Center, 975 NE 10th St, BRC 1217A, Oklahoma City, Oklahoma 73104, USA

**Keywords:** SHetA2, HPLC, Human plasma, Mouse plasma, Preclinical pharmacokinetics

## Abstract

**Background:**

SHetA2 is an oral anticancer agent being investigated for cancer treatment and prevention. The aim of this study was to develop and validate a simple, cost-effective, and sensitive HPLC-UV method for the quantification of SHetA2 in biological samples and to apply the method to pharmacokinetic studies of the drug.

**Methods:**

Sample preparation for mouse and human plasmas involved liquid-liquid precipitation and extraction using chilled acetonitrile with 2, 3-Diphenylquinoxaline as an internal standard. The separation of SHetA2 and internal standard was achieved via Waters XBridge™ BEH 130 C18 (3.5 μm, 2.1×150 mm) column coupled with a Waters XBridge™ C-18 (3.5 μm, 2.1×10 mm) guard column using 65% v/v acetonitrile: distilled water as a mobile phase in an isocratic mode with a flow rate of 0.18 ml/min. The analytes were eluted at a detection wavelength of 341 nm at a column temperature of 25°C.

**Results:**

The method was validated across a range of 5-1000 ng/ml for SHetA2 in plasma, with a lower limit of quantification of 5 ng/ml. The method showed high recovery in human (79.9-81.8%) and mouse (95.4-109.2%) plasma with no matrix effect. The intra- and inter-day accuracy and precision studies demonstrated that the method was specific, sensitive, and reliable. Stability studies showed that SHetA2 is stable for 20 h postoperatively in the auto sampler, and for six weeks at -80°C in plasma. Repetitive freezing and thawing may be avoided by preparing the aliquots and storing them at -80°C. The developed method was successfully applied to study the plasma pharmacokinetics of SHetA2 in tumor-bearing nude mice after intravenous and oral administration.

**Conclusion:**

A novel method for quantifying SHetA2 in mouse and human plasmas has been validated and is being applied for pharmacokinetic evaluation of SHetA2 in tumor-bearing mice. The developed method will be utilized for the quantification of SHetA2 in clinical studies.

## Introduction

**Figure 1:**
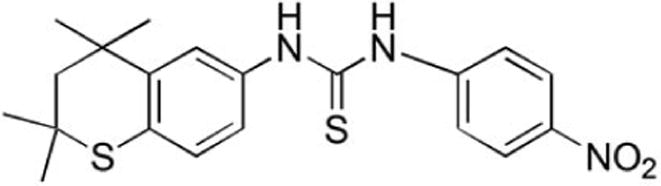
Structure of SHetA2.

SHetA2, [[(4-nitrophenyl) amino][2,2,4,4-tetramethyl thiochro-man-6-yl) amino] methane-1-thione] (Figure 1) [1] is a sulfur heteroarotinoid anticancer drug that belongs to the class of flexible heteroarotinoids (Flex-Hets), functions independent of the retinoic acid receptors, and causes potent induction of apoptosis in cancer cells without harming normal cells [2]. Originally, Flex-Hets were derived from retinoic acid, including natural retinoic acid, after a series of structural modifications. Development of retinoids as chemotherapeutic agents was hampered due to potential local and systemic toxicities, and since chronic treatment caused teratogenicity and toxicities of the skin, mucous membranes, hair, eyes, GI system, liver, endocrine system, kidneys, and bone, associated with activation of nuclear retinoid receptors. Retinoids were modified to arotinoids, which express high cytotoxic potential, but also exhibit higher toxicity. Subsequent structural modifications were carried out by incorporation of one hetero-atom (O, N, and S) in the cyclic ring of the arotinoids to block their oxidation to toxic metabolites, which resulted in the formation of heteroarotinoids with reduced toxicities. These rigid heteroarotinoids were further modified by a variety of substitutions that conferred receptor selectivity. Introducing a thiourea linker between the two heteroatoms increased structural flexibility and significantly increased the anticancer activity without activating the retinoid receptors [2,3]. Among these flexible heteroarotinoids, SHetA2 [2-5] demonstrated the greatest potency against all 60 cancer cell lines in an NCI human tumor panel [5].

SHetA2 disrupts mortalin binding to its client proteins, resulting in p53 translocation to the mitochondria and nucleus, Bcl-2 degradation, and release of p66shc to generate reactive oxygen species [6]. SHetA2 also induces G1 cell arrest and apoptosis in human ovarian cancer cells, regardless of histology, with an IC_50_ of ∼0.37-4.6 μM for growth inhibition in the National Cancer Institute 60 cell line screen[5], and inhibits the growth of ovarian xenograft tumors [2,3,5,7-10]. Extensive preclinical testing revealed that SHetA2 lacked mutagenicity, carcinogenicity, and teratogenicity [11,12]. SHetA2 has a wide therapeutic window with a No-Observed-Adverse-Effect level (NOAEL) of >1500 mg/kg/day in a 28-day dog toxicity study [13]. These tumor-selective activities and broad safety profile make SHetA2 an ideal drug for cancer prevention [6]. With this combination of properties, SHetA2 is being developed to prevent and treat different cancers, regardless of their histology.

SHetA2 is highly hydrophobic and has low oral absorption with poor bioavailability (<1%) in rats [13]. However, the bioavailability in dogs was improved with a formulation of SHetA2 suspended in 30% aqueous Kolliphor HS 15 [13]. To determine whether oral administration of SHetA2 can achieve physiological concentrations sufficient for target modulation, a phase 0 clinical study is currently underway. Phase 0 trials represent a means of accelerating drug development to assess the feasibility for further clinical development of investigational agents prior to traditional phase 1 trials [14].

Very few methods have been reported for detection of SHetA2 in the biological matrix. Zhang et al. [15] reported an HPLC-UV method for mouse plasma, requiring a multi-step sample preparation procedure. This method requires a high sample volume (200 μL) that may be limited in case of small animals for serial and repeated measurements for pharmacokinetic studies. The other method with LC/MS/MS was used for identification/qualification of its metabolites in liver microsomes and pre-clinical animals [25], but not for quantification purposes. Currently, there is no available method for quantification of SHetA2 in human plasma. Although the preclinical pharmacokinetics of SHetA2 have been previously reported [13,15], those studies were performed in healthy animals at much higher than therapeutic doses to evaluate the toxicokinetics. A better understanding of the drug's pharmacokinetics in relevant disease models, such as tumor-bearing mice, is needed to link drug exposure to treatment efficacy. Thus, a reliable, sensitive bioanalytical method for quantifying SHetA2 is required for characterization of its pharmacokinetic properties, pre-clinically and clinically.

The present study describes the development and validation of an HPLC-UV bioanalytical method to quantify SHetA2 in human and mouse plasma. The results showed that our method was sensitive, accurate, and reliable; the method has been successfully applied to the study of the pharmacokinetics of SHetA2 in orthotopic tumor-bearing nude mice.

## Materials and methods

### Chemicals and reagents

SHetA2 was provided by the US National Cancer Institute RAPID Program. The internal standard (2, 3-Diphenylquinoxaline) was purchased from Acros Organics (New Jersey, US). HPLC-grade acetonitrile (HPLC-JT9012) was obtained from VWR (Pennsylvania, US). Mouse plasma (Na heparinized) from Balb C mice was procured from Innovative Research (Michigan, US). Human plasma was purchased from Oklahoma Blood Institute (Oklahoma City, US). All plasmas were stored at -20°C. All mobile phases and standards were prepared using distilled water prepared with a Millipore (Milford, US) distillation apparatus.

### HPLC apparatus and conditions

The chromatographic apparatus consisted of an Agilent 1260 HPLC system equipped with a binary pump (Agilent model G1312C), VWD UV detector (G1314F) set at 341 nm, standard autosampler with thermostat (G1329B), online vacuum degasser (G4225A), and column head (G1316A). Chromatographic separation was carried out using a Waters XBridge™ BEH 130 C18 (3.5 μm, 2.1×150 mm) column coupled with a Waters XBridge™ C-18 (3.5 μm, 2.1×10 mm) guard column maintained at 25°C. The analysis was performed at a column temperature of 25°C using a mobile phase of acetonitrile (ACN) and distilled water (65:35, v/v), an isocratic mode, pumped at a flow rate of 0.18 ml/min.

2, 3-Diphenylquinoxaline, which is structurally unrelated but has a reasonable absorbance at detection wavelength, was used as an internal standard for the determination of SHetA2 in plasma. The sample (70 μl) was injected into the HPLC system through the autosampler, which was maintained at 4°C through the thermostat and eluted for a run time of 40 min. All biological samples were filtered using 0.2-μm polypropylene Captiva ND plates, (Part # A5969002, Agilent Technologies), 1-ml Captiva 96-deep well collection plates (Part # A696001000, Agilent Technologies), and a CaptiVac vacuum collar (Part # A796, Agilent Technologies).

### Preparation of standard solutions

A master stock solution of 100 μg/ml of SHetA2 was prepared in 100% ACN and was stored in amber glass vials at -20°C. Serial dilutions were prepared from this master stock solution for a calibration curve of the standard solutions ranging from 2.5 to 1000 ng/mL in 50% v/v ACN: water. The internal standard master stock solution of 100 μg/ml was prepared in 100% ACN and stored at -20°C. The internal standard was diluted with ACN to prepare 5 μg/mL of working standard solution.

### Preparation of samples

#### Spiked Plasma Standards (Calibration Curve)

At the time of analysis, the mouse and human plasmas were thawed under ice and spiked with a100 μg/ml master stock solution of SHetA2 (Stock Vial #A) prepared in ACN and then diluted with 225 μl of the respective blank plasma to prepare a 10 μg/ml mixture (Stock Vial #B). This stock Vial #B was used for the preparation of the calibration curve, as shown in Table 1. The calibration curve in human and mouse plasma was constructed at concentrations ranging from 5 to 1000 ng/ml.

#### Quality control samples

Quality control (QC) samples were prepared in mouse and human plasma to assess the precision and accuracy of the bioanalytical method, stability of SHetA2, and recovery in the respective matrixes. Three levels of QC samples were prepared such that the low QC was about three times the lower limit of quantification (LLOQ), the middle QC was in the midrange, i.e., at about geometric mean of the low and high QC concentrations, and the high QC was near the high end of the range, ∼70-85% of the upper limit of quantification (ULOQ) [16,17].

### Extraction procedure

Twenty microliters of the internal standard working solution (5 μg/ml) were added to an amber-colored eppendorf tube kept on ice, followed by 180 μl of spiked plasma standards, QCs, or unknown plasma sample. The mixture was vortexed vigorously for 1 min and precipitated using chilled ACN (160 μL), followed by vigorous vortexing for 10 min. The mixture was centrifuged at 21,381× *g* for 15 min at 4°C. The supernatant was collected and filtered by Captiva filtration by applying a vacuum of not more than 5 inches of Hg. The filtrate was then injected (70 μl) and analyzed using HPLC/UV. For mouse sample preparation, the volume of spiked plasma standards, QCs, and unknown plasma samples was halved (90 μl), as were the internal standard (10 μl), and chilled ACN (80 μl), but the injection volume was the same. In our method, the required sample volume for mouse plasma is less than half that of the previously reported method [15].

### Optimization of experimental parameters

Various experimental parameters, including mobile phase composition, flow rate, detection wavelength, internal standard, and the column temperature, were optimized for the analysis of SHetA2 and internal standard using a reverse phase-HPLC/UV system in an isocratic mode.

Liquid-liquid extraction of SHetA2 and internal standard from plasma were also tested using various organic solvents, including acetonitrile, chloroform, and mixtures of acetonitrile with 1-chlorobutane or chloroform. The selection of protein precipitation and extraction method was based on maximum recovery of both analytes and selectivity.

### Selection of stationary phase

Various analytical columns, such as the 201TP52 201TP TM C-18 column (5 μm, 2.1 mm×250 mm) from Vydac, the SB-C-18 Solvent Saver column (5 μm, 2.1mm×250 mm) from Agilent, and the Xbridge BEH C-18 Column (3.5 μm, 2.1mm×150 mm) with the XBridge C-18 guard column (3.5 μm, 2.1 mm×10 mm) from Waters, were tested to optimize the separation, sensitivity, and selectivity for SHetA2 and internal standard in plasmas.

### Mobile phase

Different compositions of acetonitrile and distilled water ranging from 40 to 80% v/v were investigated to maximize the separation, sensitivity, and peak resolution.

### Flow rate

SHetA2 and selected internal standard were analyzed with different flow rates in a range of 0.1-0.3 ml/min to identify the best sensitivity and resolution of the target peaks.

### Column and autosampler temperature

The temperature of the column head was varied from 20°C to 30°C to study its effect on the chromatograms of analytes by comparing the sensitivity, retention time, separation, and peak resolution of SHetA2 and internal standard. The temperature of the autosampler was kept at 4°C to minimize any degradation while samples were queued for the analysis.

### Selection of detection wavelength

For simultaneous measurement of SHetA2 and internal standard, the analytes were studied in a wavelength range from 250 to 750 nm using UV-spectroscopy. The wavelength that gave good peak resolution and best sensitivity was selected.

### Selection of internal standard

Different compounds, including quizalofop ethyl, quizalofop methyl, fluazifop-butyl, propaquiafop, 6,7-dimethyl-2,3-di (2-pyridyl) quinoxaline,2, 3-diphenyl-5,6-benzoquinoxaline, and 2, 3-diphenylquinoxaline, were assessed for their applicability as internal standards. All of the above compounds were compared for their reasonable absorbance and sensitivity at maximum absorbance of SHetA2.

### Method validation

The method was validated to ensure that it is reliable, reproducible, and of good quality. Full validation for this bioanalytical method was conducted and involved analyzing linearity (standard curve), accuracy, precision (repeatability [within-day] and ruggedness [between-day]), specificity, recovery, sensitivity, and stability. In addition, matrix effects and carry-over were investigated.

### Linearity

Per FDA guidelines, a linear range should contain at least six-to-eight concentrations (excluding blank) using single or replicate aliquots [17]. The standard curve was freshly prepared for each assay. Per FDA guidelines, the variation in back-calculated values should not exceed ±15%, except LLOQ (±20%). The acceptance criteria for the standard curve were such that at least 75% of standards, including the LLOQ, and 67% of the QC samples (low, medium, and high) should meet the above limits [18]. Linearity was assessed from the calibration curves constructed by plotting the response ratios (ratio of peak areas of the analytes to internal standard) with respect to concentrations of analyte (5-1000 ng/ml) using linear least-squares regression. The resulting plots were characterized by the slope (m), intercept (b), correlation coefficient (*r*), and covariance (%RSD) using the regression equation in Microsoft Excel 2010.

### Sensitivity and specifcity

The sensitivity of the bioanalytical method was evaluated by quantifying the lower limit of detection (LLOD) and lower limit of quantification (LLOQ) for SHetA2. The LLOD of SHetA2 is the concentration at which the signal-to-noise ratio (S/N) is 3, and LLOQ is the minimum concentration of analyte that can be determined with S/N of 10.

Sensitivity analysis was carried out using the calibration curve in plasmas. The LLOQ was determined by measuring the analyte response with a precision of <20%. Specificity of the bioanalytical method was assessed qualitatively for the presence of interfering peaks and changes in the retention time by comparing chromatograms of extracts of multiple lots of blank human/mouse plasma with plasmas containing spiked internal standard and SHetA2. The chromatographic system was also checked for injection carry-over.

### Accuracy and precision

The accuracy of any bioanalytical method depends on the closeness between the observed and the true value of concentration, expressed either as % bias or % nominal, and is determined using QC samples [19]. Accuracy is measured using five determinations per concentration. The accuracy was calculated according to Eq. (1) [20].

(1) $$\text{Accuracy}=\frac{\text{Observed concentration}}{\text{Normal concentration}}\%$$

The intra- and inter-day precision of the proposed method was tested by intra- and inter-day analyses of five replicates of each QC. The relative standard deviation (%RSD) of each calculated concentration was used as a measurement of precision.

### Extraction recovery

The recoveries of SHetA2 and internal standard throughout the extraction procedure were determined by comparing the responses of the analytes extracted from five replicates of three QCs with the responses of the analytes in the un-extracted standard solutions at equivalent concentrations. Recoveries were determined at three concentrations for SHetA2 and at a single concentration for the internal standard.

### Stability study

The stability of SHetA2 in mouse and human plasma was analyzed under different conditions. The QC samples (*n*=5) in three concentrations were analyzed for autosampler stability, for long-term stability for six weeks, and three freeze-thaw cycles at -80°C, thawed at 4°C. Deterioration of each analyte was defined as a greater than 15% relative error (RE) calculated, according to Eq. (2) SHetA2 in human and mouse plasmas [21,22].

(2) $$\%\text{RE}=\frac{\text{Observed concentration}-\text{Normal concentration}}{\text{Normal concentration}}\%100$$

### Pharmacokinetic study application

The developed bioanalytical method was applied to a pharmacokinetic study of SHetA2 in tumor-bearing female athymic nude mice (Crl: NU(NCr)-Foxn1nu strain code 490 homozygous, Charles River Laboratories). Doses of 10 mg/kg for intravenous and 60 mg/kg for oral administration were selected based on the previous reports in which SHetA2 reduced the growth of ovarian and kidney cancerous xenograft tumors at a dose range of 10-60 mg/kg/day [5,23]. All procedures were performed according to a protocol (14-139-NSHIC) in compliance with Institutional Animal Care and Use Committee (IACUC) of the University of Oklahoma Health Sciences Center.

Nude mice (6-8 weeks old, 15-20 g) were housed under constant temperature, humidity, and lighting (12 h light per day) at the animal facility and were allowed free access to food and water. One million SKOV3-luc human ovarian cancer cells (a generous gift from Dr. Anil Sood, MD Anderson Cancer Center, Houston, TX) suspended in 100 μl of Hank's Balanced Salt Solution were injected intraperitoneally (i.p.). Tumor growth and development was monitored by bioluminescent imaging performed with the Care stream XTREME imaging system to visualize peritoneal tumors. For imaging purposes, 125 μl of D-luciferin (30 mg/ml; Caliper Life Sci. Inc., Hopkinton, MA) was injected i.p. into each mouse (20-25 g). Mice were then placed in an isoflurane chamber (2%, 1 l/min) for 15 min and transferred to the imaging chamber. At the end of three weeks, the animals were randomized based on luminal intensity. For intravenous (i.v.) administration, 2.5 mg/ml of SHetA2 was prepared in 10% Kolliphor HS 15: sterile PBS v/v and was filtered by Captiva filtration. Tumor-bearing mice were randomized into seven groups of three mice and received 10 mg/kg SHetA2 solution i.v. *via* eye vein (100 μl adjusted by body weight). Three mice were euthanized at 0.25, 0.5, 1, 2, 4, 18, and 24 h after dosing. Blood was collected from the inferior venacava under deep isoflurane anesthesia. Two additional blood samples were collected at 0.08 and 12 h after dosing *via* a saphenous vein (0.2 ml) under appropriate restraint. For oral (p.o.) administration, SHetA2 was prepared in 30% Kolliphor with a final concentration of 6 mg/ml to provide an oral dose of 60 mg/kg when administered p.o. with a dosing volume of 10 ml/kg. Tumor-bearing mice were randomized into fourteen groups of three mice and were given a single dose of the above formulation. Three mice were euthanized at 0.25, 0.5, 1, 1.5, 2, 2.5, 3, 4, 6, 8, 12, 24, 36, and 48 hours after dosing. Blood was collected into heparinized tubes from the inferior venacava under deep isoflurane anesthesia. All blood collection procedures were performed under a biosafety cabinet per IACUC guidelines. Plasma was collected by centrifuging at 1200x *g* for 10 min at 4°C and was stored at -80°C until the bioanalysis.

Plasma concentration-time data from both i.v. and p.o. doses were simultaneously fitted to a two-compartment pharmacokinetic model using the Phoenix WinNonlin (Version 6.4, Pharsight, CA), and relevant pharmacokinetic parameters were obtained.

## Results and Discussion

The developed bioanalytical method is rapid, sensitive, reproducible, and easy to automate for the determination of SHetA2 in human and mouse plasma using 2, 3-diphenylquinoxaline as an internal standard. Various experimental parameters and chromatographic conditions were optimized as per the standard guidelines [24]. We observed good separation with good response in a run time of 15 min for standard mixtures and 40 min for plasma samples.

### Chromatographic conditions and sample preparation

Various experimental parameters were compared to select the optimum stationary phase, mobile phase, flow rate, column temperature, internal standard, and detection wavelengths. Best separations between SHetA2 and internal standard were achieved using a Xbridge BEH C-18 column (3.5 μm, 2.1 mm×150 mm) with a Xbridge C-18 guard column (3.5 μm, 2.1 mm×10 mm), with no interference and good sensitivity. Different mobile phases [15,25] were studied, and 65% ACN: distilled water v/v provided good separation of SHetA2 and internal standard in both human and mouse plasma without any interference. For bioanalysis, plasma sample preparation procedures, such as solid phase extraction and liquid-liquid extraction, were applied, but these methods failed to remove interferences at the SHetA2 retention time in chromatograms of blank human plasma. A simple protein precipitation with chilled acetonitrile followed by Captiva filtration provided interference-free elution of SHetA2 and internal standard in both human and mouse plasma with good sensitivity and reproducibility, which makes our method time-efficient and cost-effective. To select a detection wavelength, a solution of SHetA2 in ACN was scanned using a double beam UV spectro-photometer. The scan showed maximum absorbance at 341 nm, which was selected as the working wavelength for the detector of HPLC. Various internal standards were studied based on their absorbance at 341 nm. Among all internal standards studied, 2,3-diphenylquinoxaline showed good separation from SHetA2 with reasonable sensitivity and good recovery for the protein precipitation method. Other internal standards either interfered with the SHetA2 retention time or eluted from the column after long run time (<60 min). The entire bioanalytical method was then optimized for a flow rate of 0.18 ml/min to have a good separation without compromising for run time and sensitivity. Based on previous stability recommendations [15], the column temperature and autosampler temperature were kept at 25°C and 4°C, respectively.

**Figure 2:**
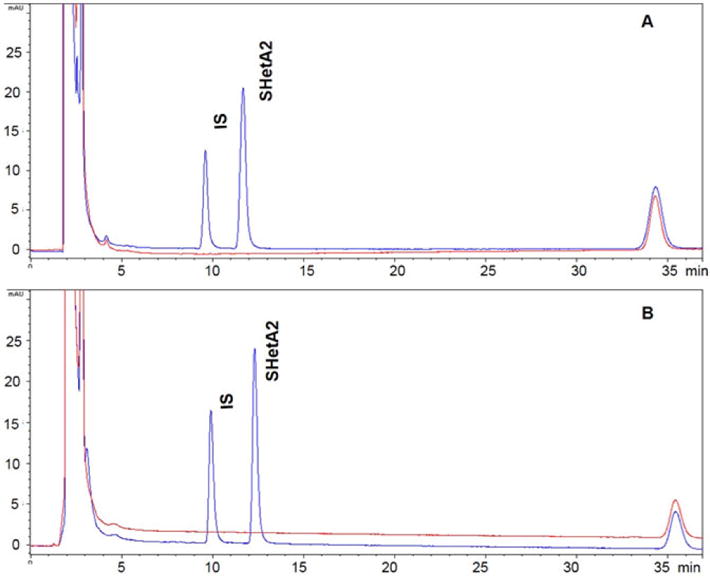
Representative chromatogram of human (**A**) and mouse (**B**) plasma spiked with SHetA2 (1000 ng/ml) and internal standard (500 ng/ml). An internal standard (IS) and SHetA2 were eluted at 9.5 and 11.6 min, respectively, in human plasma, and 9.8 and 12.3 min, respectively, in mouse plasma (blue). No interference was observed with blank plasmas (red) at the retention times of interest.

All above conditions were verified by spiking SHetA2 in human (Figure 2A) and mouse (Figure 2B) plasma at a final concentration of 1000 ng/ml, and were analyzed by HPLC with a diode array detector set at 341 nm.

### Method validation

The developed bioanalytical method was validated according to the standard guidelines for selectivity, sensitivity, recovery, precision, and robustness.

### Linearity

The linearity of the method was determined from the calibration curve of the standard mixtures and spiked plasma samples. Calibration curves were constructed at concentrations ranging from 5 to 1000 ng/ml for SHetA2 for standard mixtures and spiked plasmas, in both mouse and human plasma. The method was linear within the concentration range for both human and mouse plasma. Calibration curves of SHetA2 were prepared by least-squares linear regression and linear over the concentration range in human (*y*=0.0014*x*–0.0007, r^2^=0.999) and mouse (*y*=0.0015*x*+0.0038, r^2^ =0.999) plasma. Peak area response (PAR) ratios of SHetA2 to the internal standard were used as the response measure for the calibration curves. Linearity was also confirmed by the back-calculated calibrator concentrations.

### Sensitivity and specificity

**Figure 3:**
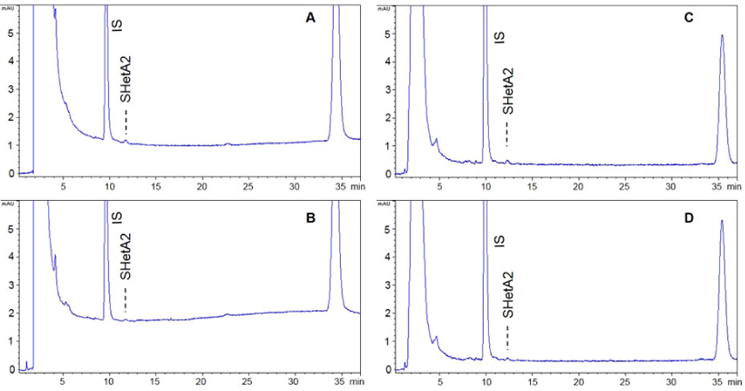
Representative chromatograms of SHetA2 at LLOQ (5 ng/ml; **A** and **C**) and LLOD (2.5 ng/ml; **B** and **D**) in human (*left panels*) and mouse (*right panels*) plasma.

Spiked plasma showing LLOQ (5 ng/ml) and LLOD (2.5 ng/ml) were compared with the respective human and mouse blank plasma (Figure 3). The LLOQ for human (%RSD=7.0 and % Ac-curacy=102.7) and mouse (%RSD=12.3 and % Accuracy=85.3) plasma were precise and accurate. The limit of detection and limit of quantification of SHetA2 with the current method were better than the previously reported method with the LLOQ of 10 ng/ml [15].

Specificity was confirmed by the absence of any interfering peaks in different lots of human and mouse plasmas at the retention times of SHetA2 and internal standard. The carryover effect was not observed for the current method. The lack of carry-over effect was confirmed by the absence of a peak in the blank plasma following the highest standard in the calibration curve or the highest quality control.

### Precision and accuracy

The results of accuracy and precision measurements were analyzed using three QCs (10, 100, and 800 ng/ml) in plasmas (*n*=5), as shown in Table 2. Both the intra- and inter-day precision (%RSD) in both matrices were less than 15%. The intra-day accuracy ranged from 100.5 to 110.2%, and inter-day accuracy ranged from 96.9 to 101.2% in mouse plasma. In human plasma, intra-day accuracy ranged from 99.4 to 109.1% and inter-day accuracy ranged from 102.6 to 112.0%. The results indicated that the developed method in this study has satisfactory accuracy, precision, and reproducibility.

### Extraction recovery

Extraction recovery of SHetA2 from human and mouse plasma was studied at 10, 100, and 800 ng/ml by comparing the peak areas between extracted plasma and standard mixtures. The extraction recovery was 79.9-81.8% for human plasma and 95.4-109.2% for mouse plasma. The recovery of internal standard was 86.0% and 99.96% for human and mouse plasma, respectively. Such a difference in recovery may be attributed to protein binding of drugs among different species [26].

### Sample stability

The stability of SHetA2 under various conditions was tested at three QCs for autosampler, long-term, and freeze-thaw cycle in human and mouse plasma. The concentrations of all of the stability samples were compared with the mean of the back-calculated values for the standards at the appropriate concentrations from the first day of the long-term stability testing [17]. The stability of the processed samples during their resident time (20 h) in the autosampler was determined. There was no significant degradation (<15% RE) of the processed samples over the period of 20 h in the autosampler at 4°C, as shown in Table 3.

The long-term stability was evaluated by storing the QC samples at -80°C for six weeks. This temperature was selected after we observed 14% degradation of SHetA2 in human plasma when stored for one week at -20°C. When stored at -80°C, there was no significant degradation of the analyte over the course of six weeks, as shown in Table 4.

To study the freeze-thaw stability, QC samples were stored at -80°C and subjected to three freeze-thaw cycles under ice. After every cycle, all three QCs were analyzed and an average of the results (*n*=5) was taken, as shown in Table 5. For human plasma, a detailed analysis was carried out after each cycle. For mouse plasma, the QCs were analyzed after the third cycle. SHetA2 was stable in mouse plasma until the third freeze-thaw cycle, whereas SHetA2 in human plasma showed degradation in the third cycle, as evidenced by >15% relative error with high variability. Thus, it will be optimal to aliquot the human plasma samples from clinical trials, store them at -80°C, and avoid repeated freezing and thawing in the case of reanalysis.

### Pharmacokinetic study application

**Figure 4:**
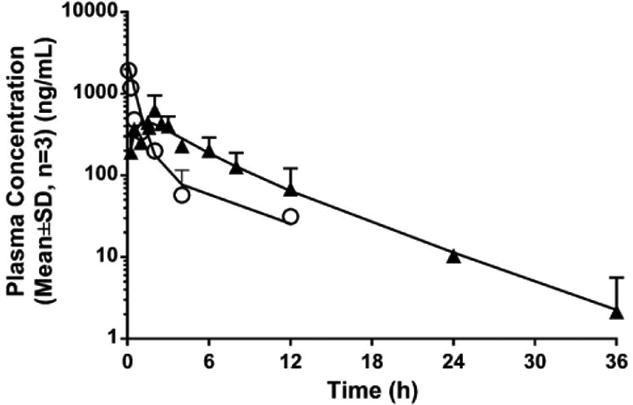
The observed (symbols) and model-predicted (lines) plasma concentration-time profiles of SHetA2 in SKOV-3-luc tumor-bearing nude mice after 10 mg/kg intravenous (○) and 60 mg/kg oral (▲) administration of SHetA2.

The proposed method was used to analyze plasma samples obtained from orthotopic tumor-bearing nude mice after intravenous (10 mg/kg) and oral administration of SHetA2 (60 mg/kg). The peak area response ratios of SHetA2 were interpolated on the mouse calibration curves to obtain the mean plasma concentration-time curves. Plasma concentration-time data from both i.v. and p.o. routes were simultaneously fitted to a two-compartment model using the Phoenix WinNonlin program and the estimated pharmacokinetic parameters, including clearance (*CL*), the volume of distribution for central (*Vc*) and peripheral (*Vp*) compartment, first-order absorption rate constant (*k_a_*), and oral bioavailability (*F*), are shown in Table 6. After 10 mg/kg i.v. administration, plasma concentration reached 1937.3 ng/ml (samples at 5 and 15 min were analyzed after 2-fold dilution) in 5 min and declined bi-exponentially, exhibiting a two-compartmental disposition (Figure 4).

Plasma SHetA2 concentration increased rapidly after oral administration and reached a peak of 628 ng/ml at 2 h. Oral bioavailability at 60 mg/kg in tumor-bearing mice was 22.6%. SHetA2 showed extensive tissue distribution (unpublished data) or tissue binding, as indicated by the large volume of distribution in tissue compartments. This is supported by the fact that this drug has an experimental log P=4.23±1.35, which indicates that it possesses the characteristics of Biopharmaceutics Classification System (BCS) class 2 drugs with high permeability and low solubility. Such drugs also undergo extensive metabolism, as characterized by Biopharmaceutics Drug Distribution and Classification System (BDDCS) Class 2 drugs [27]. Metabolic stability studies for SHetA2 are planned for a more quantitative understanding of involved cytochrome P450 (CYP) isoenzymes. The terminal elimination half-life was 4.6 h. SHetA2 was quantifiable up to 24 h (10.45 ng/ml) and detected up to 36 h (2.15 ng/ml) after oral administration, but was undetectable at 24 and 36 h after intravenous administration.

## Conclusions

In this study, we developed a simple, sensitive, cost-effective HPLC-UV method for determination of SHetA2 in human and mouse plasma. The developed method was validated for its specificity, accuracy, precision, sensitivity, and stability. This method was successfully applied to the pharmacokinetic study of SHetA2 in tumor-bearing nude mice to evaluate the disposition and absorption kinetics of SHetA2. In the future, this method will be utilized to determine SHetA2 concentrations in clinical samples from planned Phase 0 clinical trials. The method could be modified to detect and analyze various SHetA2 analogs in the pipeline and will be applied to study the tissue distribution of SHetA2 in different species.

## Figures and Tables

**Table 1:** Preparation of spiked plasma calibration standards.

Final Concentration, ng/ml	Volume of Spiked Plasma, μl (Stock Vial Used)	Volume of Blank Plasma, μl	Stock Vial
5	200 (I)	200	J
10	50 (F)	450	I
25	200 (G)	200	H
50	200 (F)	200	G
100	50 (C)	450	F
250	200 (D)	200	E
500	200 (C)	200	D
1000	50 (B)	450	C

**Table 2:** Precision, accuracy, and recovery of SHetA2 in human and mouse plasmas (*n*=5).

Biological Matrix	QC (ng/ml)	Inter-day precision			Intra-day precision			Recovery (Mean±%RSD)

		Mean	RSD (%)	Accuracy (Mean, %)	Mean	RSD (%)	Accuracy (Mean, %)	
Human plasma	10	11.2	7.3	112.0	10.9	13.3	109.1	79.9±8.9
	100	110.2	9.8	110.2	106.3	11.3	106.3	81.4±11.0
	800	820.6	3.3	102.6	794.9	12.5	99.4	81.8±11.0

Mouse plasma	10	9.7	12.3	96.9	9.0	13.4	110.2	95.4±6.2
	100	99.7	4.2	99.7	97.2	12.9	102.8	100.4±2.2
	800	809.4	3.4	101.2	795.9	13.0	100.5	109.2±13.6

**Table 3:** Autosampler stability of SHetA2 in human and mouse plasmas.

Biological matrix	QC (ng/ml)	Mean	RE (%)	RSD (%)
Human plasma	10	9.5	-5.1	11.7
	100	103.1	3.1	7.2
	800	719.5	-10.1	10.4

Mouse plasma	10	8.6	7.0	9.8
	100	105.9	5.9	7.2
	800	855.9	-13.5	0.6

**Table 4:** Long-term stability of SHetA2 in human and mouse plasmas at −80°C.

Biological Matrix	QC (ng/ml)	Week 4			Week 6		

		Mean	RE (%)	RSD (%)	Mean	RE (%)	RSD (%)
Human plasma	10	11.9	0.09	12.5	12.7	7	13.2
	100	114.3	-1.7	11.3	106.9	-8	13.5
	800	755.9	-14.3	4.8	863.4	-2	4.8

Mouse plasma	10	11.7	6.5	8.5	9.5	-13.3	3.7
	100	115.1	12.4	5.4	108.4	5.9	1.3
	800	888.6	5.5	1.6	889.0	5.6	1.5

**Table 5:** Multiple freeze-thaw cycle stability of SHetA2 in human and mouse plasmas at −80°C.

Biological Matrix	QC (ng/ml)	First Cycle			Second Cycle			Third Cycle		

		Mean	RE (%)	RSD (%)	Mean	RE (%)	RSD (%)	Mean	RE (%)	RSD (%)
Human plasma	10	10.5	-10.3	13.2	10.2	-12.8	5.7	13.2	-36.7	19.4
	100	91.0	-4.1	14.3	87	-8.3	10.9	57.1	-39.8	12.9
	800	694.2	-9.7	3.8	657.8	-14.4	3.4	486.8	12.8	2.0

Mouse plasma	10	--	--	--	--	--	--	8.4	10.2	-11.3
	100	--	--	--	--	--	--	88.8	2.7	-13.3
	800	--	--	--	--	--	--	762	4.3	-9.5

**Table 6:** Pharmacokinetic parameters of SHetA2 in tumor-bearing nude mice.

Parameter	Estimate (%RSD)
CL (L/h/kg)	4.5 (11.8)
Vc (L/kg)	4.1 (20.4)
Vp(L/kg)	10.8 (52.1)
k_a_ (h^-1^)	0.3 (19.1)
F (%)	22.6 (19.1)

## List of abbreviations

LLOQ: Lower limit of quantification
LLOD: Lower limit of detection
SHetA2: [[(4-nitrophenyl)amino][2,2,4,4-tetramethyl thiochro-man-6-yl)amino] methane-1-thione]
Flex-Hets: Flexible heteroarotinoids
NOAEL: No-Observed-Adverse-Effect level
IC_50_: Concentration producing 50% maximum inhibition
%RE: Percentage relative error
%RSD: Percentage relative standard deviation
